# Liver Transplantation for Alcohol-Related Liver Disease (ARLD): An Update on Controversies and Considerations

**DOI:** 10.1155/2020/8862152

**Published:** 2020-09-17

**Authors:** Dipesh Kumar Yadav, Qi Zhang, Xueli Bai, Enliang Li, Tingbo Liang

**Affiliations:** ^1^Department of Hepatobiliary Surgery & Liver Transplantation, The First Affiliated Hospital, Zhejiang University, Hangzhou 310003, China; ^2^Zhejiang Provincial Key Laboratory of Pancreatic Disease, Hangzhou 310003, China; ^3^Innovation Center for the Study of Pancreatic Diseases, Hangzhou 310003, China; ^4^Zhejiang Clinical Research Center of Hepatobiliary and Pancreatic Disease, Hangzhou 310003, China

## Abstract

According to the recent data from the United Network for Organ Sharing database, alcohol-related liver disease (ARLD) accounts to be the most common indication of liver transplantation (LT) waiting lists in the United States among men without hepatocellular carcinoma (HCC). Severe alcoholic hepatitis (AH) is serious and the life-threatening form of ARLD and should be treated timely. However, the LT for severe AH remained to be controversial among the transplant community because of marked interests about the constrained organ supply and the hazard that the AH liver recipient will return to risky drinking. Early LT for ARLD refers for a patient with severe AH undergoing LT who are non-responder to medical treatments. These patients are generally on the existing waiting list but usually followed by 6-month duration of alcohol abstinence. However, the rule of 6-month alcohol abstinence need before the LT is ambiguous. The 6-month alcohol abstinence was consistently defended in light of the compelling fact that it would enable patients to recoup from the intense impacts of alcohol to the liver. In routine, however, the purported “6-month abstinence rule” turned into a surrogate for the forecast of future drinking by ARLD patients for the LT. Careful consideration should be given to the alcohol use disorder of craving and the hazard for recidivism after the LT. As for the current situation, there, urgently, is a specific need of standardized criteria for the evaluation of patients with severe AH for earlier LT. Moreover, further studies are required precisely to develop an accurate prediction model for posttransplant alcohol recidivism. Additionally, development of a standardized protocol for post-LT follow-up and management is further needed. We carefully outlined the published experience with the LT for ARLD in this review.

## 1. Introduction

The world's first liver transplant was done by Thomas Starzl on March 1963 in a 3-year-old boy with biliary atresia. However, the patient died during operation due to coagulation failure and uncontrolled bleeding [1]. Another 5 more liver transplantations (LTs) were performed by Thomas Starzl in the following years but none of them survived more than 23 days [1]. The discovery of cyclosporin A brought a major breakthrough in the LT outcome, where patients survived 1 to 5 years [2, 3].

The outcomes of the LT recipients have significantly enhanced throughout the years through therapeutic advances, including ameliorated surgical techniques, powerful antimicrobial treatment, and effective immunosuppressive drug regimen. However, despite the considerable improvements in results, LT is experiencing an emerging challenge of demand and supply of available organs, where patients waiting for the LT are exceedingly higher than that of the total LT performed, and most of the patients die on the waiting list. In that regard, the governing body for organ allocation works on the concept of giving donor liver first to those who are unlikely to survive without LT, and to those who are most likely to be benefited from the LT. Thus, allocating organ on the basis of fair, legal, and ethical system [4].

An assortment of explicit conditions regularly animates debate with respect to the proper candidate for the LT. We reviewed the published literature with the LT for alcohol-related liver disease (ARLD).

## 2. Alcohol-Related Liver Disease (ARLD)

ARLD consists of a number of hepatic inflammatory injury conditions due to excessive alcohol abuse, which include uncomplicated steatosis, steatohepatitis (with and/or without fibrosis), severe alcoholic hepatitis (AH), and cirrhosis [4]. Alcohol consumption remains a global problem and accounts for about 3.8% of worldwide mortality [5]. Most of the patients with ARLD suffer from nutritional deficiencies, and it is strongly suggested to offer high-calorie and high-protein diets to such patients to improve their nutritional status and survival [6].

Severe AH is serious and the life-threatening form of ARLD and should be treated timely. In addition to abstinence from alcohol and nutritional support, treatment with corticosteroids is suggested for patients with severe AH with the Maddrey discriminant function (MDF) score more than 32 [7]. Treatment with prednisolone 40 mg/day is recommended and given for 28 days if the Lille score is less than 0.45 after a week [7]. The Lille score is used to predict mortality in patients with AH who are nonresponders to steroid therapy; it includes age, albumin, initial bilirubin level, bilirubin level at day 7, creatinine level, and prothrombin time. Patients with a Lille score more than 0.45 after 1 week of corticosteroid therapy are likely nonresponders to steroid therapy and are at a high risk of death if corticosteroid therapy is continued. Moreover, a Lille score above 0.56 flags discontinuation of corticosteroid therapy and should be considered for the early LT [8].

### 2.1. Liver Transplantation for ARLD

**Figure 1:**
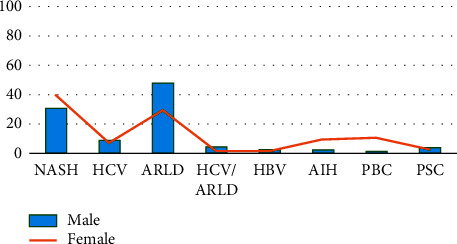
Common indications for the liver transplantation waiting list in the United States without HCC by gender in 2019 [9]. AIH: autoimmune hepatitis; ARLD: alcohol-related liver disease; HBV: hepatitis B virus; HCC: hepatocellular carcinoma; HCV: hepatitis C virus; NASH: nonalcoholic steatohepatitis; PBC: primary biliary cholangitis; PSC: primary sclerosing cholangitis.

According to the recent data from the United Network for Organ Sharing database, ARLD accounts to be the most common indication of the LT waiting lists in the United States among men without hepatocellular carcinoma (HCC), i.e., 47.7% (Figure 1) [9], thus, by exceeding hepatitis C virus (HCV) which used to be the most common indication for the LT in the United States for almost 2 decades [10, 11]. Numerous studies have shown comparable results for the survival of transplanted patients with ARLD with that of other etiologies of chronic liver disease [12, 13]. Nonetheless, published studies also suggest that patients with ARLD who continue to drink after the LT develop more severe hepatic injury leading to early graft failure and reduced patient survival. Additionally, posttransplant mortality likewise also results from the cardiovascular disease, cerebrovascular events, gastrointestinal and respiratory malignancies, and suicide [4].

### 2.2. Liver Transplantation for ARLD as the Controversy

LT for the ARLD remains fairly disputable for two primary reasons. To start with, society holds a negative impression of LT for frequent drinkers. Independent studies have uncovered that the general population and even doctors see organ allotment to the patients with ARLD, which is seen as a self-inflicted disease, less positively than those with the acquired liver disease [14].

Second, incredible concern remains with respect to the possibility of alcohol relapse after the LT, and the transplanted liver might be seen as a “squandered organ” in case of alcohol relapse after the LT [4]. This worry is magnified on account of severe AH when the times of temperance before the LT are generally far-fetched typically contrasted with higher death rates without the LT. In any case, attitudes seem to be changing, and the LT for AH is getting more accepted at recent times. It was not very long back that the AH was frequently viewed as an absolute contraindication for the LT.

### 2.3. The 6-Month Abstinence Rule

Indeed, even though the understanding of alcohol use disorder turned out to be progressively general inside the transplant community, LT as a treatment for patients with serious AH stayed “taboo.” The importance of this thought was not offering LT to those who had a short duration of alcohol abstinence or no spell of alcohol abstinence. The rule of 6-month alcohol abstinence need before the LT is ambiguous. Essentially, the 6-month alcohol abstinence was defended in light of the fact that it would enable patients to recoup from the intense impacts of alcohol to the liver [15]. In routine, however, the purported “6-month abstinence rule” turned into a surrogate for the forecast of future drinking by the ARLD patients for the LT.

From the beginning of the LT for ARLD, specialists in addiction medicine were not convinced by the 6-month abstinence rule. In fact, some studies recommended that restraint in the men with ARLD was reliable only after five years [16]. Beresford et al. suggested that the patients with ARLD should be evaluated by an addiction expert before undergoing LT [17]. In recent years, several protocols and prognostic tools have been proposed to assess the risk of alcohol relapse with a coordinated assessment of addiction medicine experts; and these include the University of Michigan Alcoholism Prognosis Score, Alcohol Relapse Risk Assessment (ARRA), High-Risk Alcoholism Relapse (HRAR), and Stanford Integrated Psychosocial Assessment for Transplantation (SIPAT) [13, 18].

### 2.4. In Favor of Early Liver Transplantation for ARLD

Early LT for ARLD refers for a patient with severe AH undergoing LT who are non-responder to medical treatments. These patients are generally on the existing waiting list but usually followed by 6-month duration of alcohol abstinence (Table 1) [19]. The truth of the matter is, at present, there are not many effective treatment alternatives for the severe AH. In spite of the fact that corticosteroids are prescribed as a first-line treatment for the severe AH, nonresponse to medical treatment occurs in about 40% of the patients and is related to more than 70% deaths within 6 months [20]. Patients responding to corticosteroids and remaining abstinent bear a low risk of mortality [7]. Likewise, patients who are nonresponders to corticosteroids and remain abstinent have an acceptable prognosis and might be considered for earlier LT [21]. However, numerous patients with the severe AH and nonresponders to corticosteroid treatment are at high danger of mortality regardless of abstinence. In this manner, the absence of rescue therapy for these patients is the reason for considering salvage or early LT [13].

The seminal study conducted by Mathurin et al., in 2011, included 26 patients with severe AH who were nonresponders to corticosteroids and underwent early LT. The total 6-month and 2-year survival rates were essentially higher among the patients who underwent early LT than the patients who could not undergo LT [21]. Since the reporting of this study, there has been a significant rise in cases of earlier LT for the patients with severe AH. Various other centers acknowledged this issue, with promising results [22]. Recently, in a 12-center retrospective study of 147 patients with the severe AH (median MELD score 39 and Lille score 0.82) and ahead 6 months of alcohol abstinence underwent early LT, the study revealed 1- and 3-year survival rates of 94% and 84%, respectively; these results were encouraging and comparable with the survival outcomes of the LT with the other indications of the LT [12].

In the study by Im et al., the study compared nine patients with the severe AH undergoing LT with a matched control group that were managed by medical therapy, and the study showed an excellent 6-month survival for the patients with severe AH undergoing LT (89%) compared to that of the patients in the control group (11%) [19]. Similarly, the other study reported from Johns Hopkins compared 46 patients who underwent LT for severe AH with a control group of 34 patients with alcoholic cirrhosis (AC) and 6-month abstinence transplanted during the same time interval (2012–2017) [23]. In this study, the patients with severe AH were generally comparative, yet were somewhat younger, and had a significantly higher MELD score (average MELD score above 35) and a shorter span of restraint from drinking. The study found that the 1-year survival was comparable in the two groups (97% and 100%, respectively), and recidivism was additionally comparative in both groups, i.e., 24% and 28%, respectively. The criteria for selection of the patients for the LT in this study were more flexible than those used by Mathurin et al., permitting incorporation of the patients with ongoing gastrointestinal haemorrhage. Pathology results from explanted livers showed 96% of the patients with AH exhibited cirrhosis, and 52% of explants from the patients who underwent LT for AH less than 6-month abstinence revealed pathologic features of severe AH compared to 9% of explants from the patients with more than 6-month abstinence. The gap of abstinence before the transplant was unanticipated of the survival or recidivism in either group. Another conclusion from the study is that revealed rates of recidivism in these series might remain an element of how it is characterized and how thoroughly it is sought after an observation.  Table 2 shows various studies comparing the patients with severe alcoholic hepatitis undergoing LT.

### 2.5. Alcohol Relapse after LT for ARLD and the Risk Factors

Alcohol recidivism after the LT is not phenomenal and is accounted for the development in 10% to 60% of the patients of LT for ARLD [24–26]. Despite the fact that function of the graft and survival of the patient are not influenced by periodic drinking, recidivism into risky drinking happens in around 15% to 26% of the patients [25] and is characterized as more than 20 gm/day of ethanol for the women or more than 60 gm/day of ethanol for the men decrease long-term graft function and patient survival [25]. The existence of psychotic comorbidities, young age, unmarried, unemployed, lack of social support, substance abuse, and a shorter period of abstinence before the LT are factors related with risky drinking after the LT (Table 3) [24, 27–33]. Additionally, reactivation of HCV after the LT and risky drinking after the LT are associated with the fast advancement of fibrosis [34]. Indeed, studies have revealed that 33% of the patients who prosper severe alcohol use disorder after the LT can develop cirrhosis within 5 years after the LT [26]. Likewise, recidivism of risky drinking has been related with unsatisfactory adherence to immunosuppressive drugs after the LT, which further hinders the graft function [24].

### 2.6. Follow-Up after LT for ARLD

Persistent attention should be focused regarding the management of the allograft in all LT recipients, so is for the LT recipient for ARLD; however, careful consideration, likewise, should be given to the alcohol use disorder of craving and the hazard for recidivism after the LT. Despite the fact that the LT can treat the underlying liver disease by giving the patient a physiologically functional liver that can reverse the complications related to the end-stage liver disease, it however fails to treat alcohol addiction. In this way, it is not astounding that alcohol recidivism after the LT is common. Nonetheless, some studies of best practice have outlined the long-term alcohol addiction management for the LT recipients for ARLD [26].

As mentioned earlier in this review, the patients undergoing LT for ARLD are at a long-term risk for cardiovascular disease, cerebrovascular events, gastrointestinal and respiratory malignancies, and suicide; therefore, require appropriate screening at the proper time.

## 3. Suggestions for Future Studies

As for the current situation, there is a need of standardized criteria for the evaluation of the patients with severe AH for earlier LT. Moreover, further studies are required to develop an accurate prediction model for posttransplant alcohol recidivism. Additionally, development of a standardized protocol for post-LT follow-up and management is further needed.

## Figures and Tables

**Table 1:** Commonly followed selection criteria for alcoholic hepatitis patients for liver transplantation.

Inclusion for liver transplantation
(i) Maddrey discriminant function (MDF) score >32
(ii) Lille score ≥0.45 (nonresponders to steroid therapy) or not suitable for medical management
(iii) Acute decompensated liver
(iv) Good psychosocial profile
(v) Good family and social support
(vi) Approval of the liver transplant selection committee

Exclusion for liver transplantation

(i) Poor control of infection
(ii) Comorbidity and systemic illness
(iii) Poor prognostic score (e.g., low motivation for alcohol treatment; continued alcohol use after liver disease diagnosis; and history of failed try of alcohol abstinence)
(iv) Lack of family and social support
(v) Major psychiatric disorder

**Table 2:** Studies comparing patients with severe alcoholic hepatitis (AH) undergoing liver transplantation.

Study	No. of LT for AH	Mean age at LT	Abstinence before LT (days)	MELD at the time of LT	1-year survival (%)	Return to risky drinking (%)
Mathurin et al. [21]	26	47	<90	34	77	10
Im et al. [19]	9	41	33	39	89	12.5
Weeks et al. [23]	46	50	50.5	33	97	17
Lee et al. [12]	147	43	55	38	94	11

AH: alcoholic hepatitis; LT: liver transplantation.

**Table 3:** Studies comparing risk factors for return to risky drinking for patients with alcohol-related liver disease (ARLD) undergoing liver transplantation.

Study	No. of ARLD pts	Risk factors		Return to risky drinking
		*Demographic, behavioural, and social factors*	*Comorbidity*	
Kelly et. al. [27]	100	Unmarried	Depression	10%
		Unemployed		
		Lack of social support		
		Substance abuse		
		<6-month abstinence		

Nickels et. al. [28]	27	Age < 50 years	Depression	26.9%
		Male		
		Alcohol dependence		

De Gottardi et. al. [29]	387	Age < 50 years	Psychiatric disease	11.9%
		Male		
		Unmarried		
		HRAR		
		Low SS		
		Unemployed		
		<6-month abstinence		

Karim et. al. [30]	80	Age < 50 years	Psychiatric disease	10%
		Male		
		Unmarried		
		Low SES		
		Unemployed		
		Smoking		
		Substance abuse		
		<6-month abstinence		
		Rehabilitation		

Deruytter et. al. [31]	108	Age < 50 years	Psychiatric disease	29%
		Male		
		Unmarried		
		Unemployed		
		Family history of alcohol abuse		
		Smoking		
		Alcohol dependence		

Egawa et. al. [24]	195	Male	Psychiatric disease	13.3%–50% depending upon the recipient and donor relationship
		Unmarried		
		Lack of social support		
		Unemployed smoking		
		<6-month abstinence		

Askgaard et. al. [32]	156	Male	—	18%, 24%, and 27% after 5, 10, and 15 years of posttransplant, respectively.
		Unmarried		
		Unemployed		
		Family history of alcohol abuse smoking		
		Alcohol dependence		

Wigg et. al. [33]	87	Male	Psychiatric disease	16%
		Unmarried		
		Lack of social support		
		Unemployed		
		Family history of alcohol abuse smoking		
		Substance abuse		

Satapathy et. al. [18]	241	<6-months abstinence nonalcohol-related criminal active smoking	—	10%

ARLD: alcohol-related liver disease; HRAR: high-risk alcoholism relapse; SS: docioecnomic status; and LT: liver transplantation.

## Data Availability

All the data supporting the results are shown in the paper and are available from the corresponding author upon request.
